# Phylogeny and circumscription of *Dasyphyllum* (Asteraceae: Barnadesioideae) based on molecular data with the recognition of a new genus, *Archidasyphyllum*

**DOI:** 10.7717/peerj.6475

**Published:** 2019-02-27

**Authors:** Paola de Lima Ferreira, Mariana Machado Saavedra, Milton Groppo

**Affiliations:** 1 Departamento de Biologia, Faculdade de Filosofia, Ciências e Letras de Ribeirão Preto, Universidade de São Paulo, Ribeirão Preto, São Paulo, Brasil; 2 Departamento de Botânica, Instituto de Biologia, Universidade Federal do Rio de Janeiro, Rio de Janeiro, Rio de Janeiro, Brazil

**Keywords:** Asterids, Compositae, Character Evolution, South America, Systematics, Taxonomy

## Abstract

*Dasyphyllum* Kunth is the most diverse genus of the South American subfamily Barnadesioideae (Asteraceae), comprising 33 species that occur in tropical Andes, Atlantic Forest, Caatinga, Cerrado, and Chaco. Based on distribution, variation in anther apical appendages, and leaf venation pattern, it has traditionally been divided into two subgenera, namely, *Archidasyphyllum* and *Dasyphyllum*. Further, based on involucre size and capitula arrangement, two sections have been recognized within subgenus *Dasyphyllum*: *Macrocephala* and *Microcephala* (=*Dasyphyllum*). Here, we report a phylogenetic analysis performed to test the monophyly of *Dasyphyllum* and its infrageneric classification based on molecular data from three non-coding regions (*trn*L-*trn*F, *psb*A-*trn*H, and ITS), using a broad taxonomic sampling of *Dasyphyllum* and representatives of all nine genera of Barnadesioideae. Moreover, we used a phylogenetic framework to investigate the evolution of the morphological characters traditionally used to recognize its infrageneric groups. Our results show that neither *Dasyphyllum* nor its infrageneric classification are currently monophyletic. Based on phylogenetic, morphological, and biogeographical evidence, we propose a new circumscription for *Dasyphyllum*, elevating subgenus *Archidasyphyllum* to generic rank and doing away with the infrageneric classification. Ancestral states reconstruction shows that the ancestor of *Dasyphyllum* probably had acrodromous leaf venation, bifid anther apical appendages, involucres up to 18 mm in length, and capitula arranged in synflorescence.

## Introduction

Systematics of Asteraceae (Composite) has undergone major change over the last four decades, mainly due to the insights provided by molecular data. One of the pioneering molecular studies demonstrated an inversion of 22 kb in the chloroplast genome of all Asteraceae, except for the members of subtribe Barnadesiinae, tribe Mutiseae (Jansen & Palmer, 1987). Subsequent phylogenetic studies indicated that Barnadesiinae is the sister group to the rest of the family (Bremer, 1987; Jansen et al., 1992); therefore, the subtribe was elevated to the rank of subfamily as Barnadesioideae (Bremer & Jansen, 1992).

Barnadesioideae comprises nine genera and approximately 85 species, and is restricted to South America (Bremer, 1987, 1994; Jansen et al., 1992; Panero & Funk, 2002; Funk et al., 2005, 2009; Panero et al., 2014; Panero & Crozier, 2016; Saavedra et al., 2018). Its members are characterized by the presence of axillary spines arranged at the nodes, in pairs or in fascicles, and by the presence of unbranched three-celled hairs called “barnadesioid trichomes” on the corollas, cypselae, and pappus (Cabrera, 1959; Ezcurra, 1985; Bremer & Jansen, 1992; Bremer, 1994; Urtubey, 1999; Erbar & Leins, 2000; Ulloa, Jørgensen & Dillon, 2002; Stuessy, Urtubey & Gruenstaeudl, 2009).

**Figure 1:**
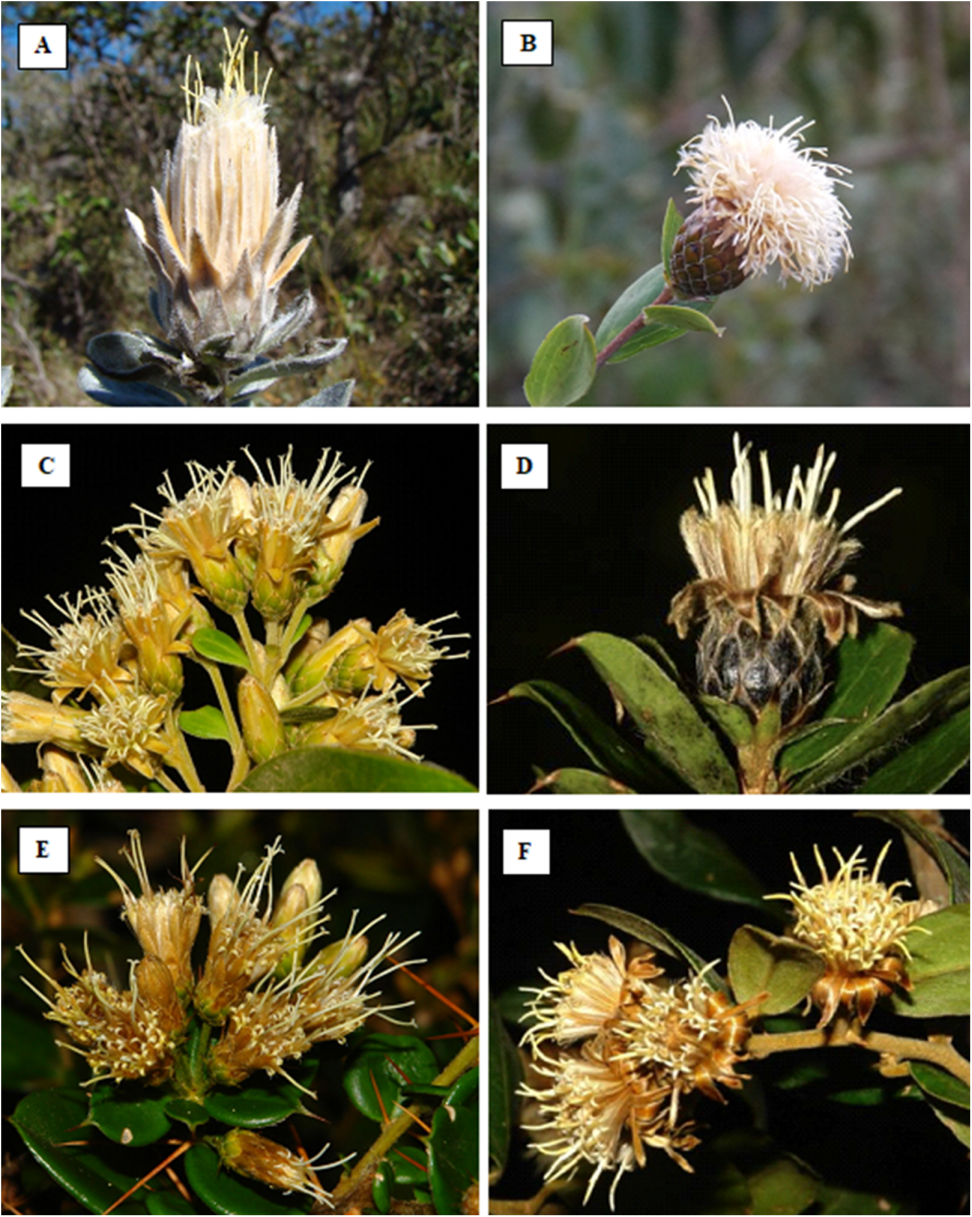
Photos of some *Dasyphyllum* species. (A) *Dasyphyllum reticulatum* (DC.) Cabrera. (B) *Dasyphyllum sprengelianum* (Gardner) Cabrera. (C) *Dasyphyllum brasiliense* (Spreng.) Cabrera. (D) *Dasyphyllum leptacanthum* (Gardner) Cabrera. (E) *Dasyphyllum diamantinense* Saavedra & M.Monge. (F) *Dasyphyllum flagellare* (Casar.) Cabrera. Photo credits: Photographs by Cláudio N. Fraga, except A (by Mariana M. Saavedra) and B (by Paola L. Ferreira).

*Dasyphyllum* is the largest genus in Barnadesioideae, comprising 33 species (Saavedra, 2011; Saavedra et al., 2018; Fig. 1) distributed from Venezuela to Northwestern Argentina, but absent in the Amazon region (Cabrera, 1959; Saavedra, 2011; Saavedra, Monge & Guimarães, 2014). The genus is morphologically diverse and can be distinguished from the other genera of Barnadesioideae by including trees, shrubs, and woody vines with pairs of straight, curved, or fasciculate spines, together with simple, alternate leaves; monoecious or gynodioecious capitula, comprising discoid heads with many types of corolla (Stuessy & Urtubey, 2006), and anthers with apical appendages that are either bifid or undivided (Cabrera, 1959; Stuessy, Urtubey & Gruenstaeudl, 2009; Saavedra, 2011).

Cabrera (1959) proposed the first infrageneric classification of *Dasyphyllum*, recognizing 36 species in two subgenera distinguished by several morphological characters and disjunct distributions. Subgenus *Archidasyphyllum* Cabrera comprised two tree-species and was characterized by the presence of leaves with pinnate venation and emarginate or obtuse anther apical appendages. Both species are restricted to the *Nothofagus* forests of central Chile and Argentina. In contrast, subgenus *Dasyphyllum* Cabrera comprised 34 tree or shrubs species, with acrodromous leaf venation and bifid anther apical appendages, distributed from the Andes eastward into tropical Argentina, Brazil, and Paraguay. Within subgenus *Dasyphyllum*, two sections are currently recognized: section *Microcephala* Cabrera (23 species) and section *Macrocephala* Cabrera (11 species). The two sections are distinguished by involucre size and capitula arrangement with section *Macrocephala* having involucre longer than 20 mm in length and arranged in a solitary or small group of heads (Figs. 1A and 1B) and section *Microcephala* having heads arranged in synflorescence (corymbiform cymes) smaller than 18 mm in length (Figs. 1C–1F).

Nonetheless, the treatment by Cabrera (1959) often relied on a single and narrow morphological concept to define the species. Due to the great morphological variation, floristic studies undertaken in Brazil have shown that many characteristics overlap; thus casting doubt on species delimitation (Roque & Pirani, 1997; Saavedra et al., 2018).

In this context, Saavedra (2011) and Saavedra et al. (2018) updated the taxonomy of *Dasyphyllum*, recognizing 33 species. Thirty of them were classified in two sections using the same morphological definition for sections provided by Cabrera (1959), that is, *Dasyphyllum* Cabrera with 24 species, and *Macrocephala* Baker ex Saavedra with six species; and the remaining three species (*D. diacanthoides, D. excelsum* belonging to *D*. subgenus *Archidasyphyllum*, and *D. hystrix*) were placed as *incertae sedis*.

Several phylogenetic studies aiming to clarify the phylogenetic relationships within Barnadesioideae have included species of *Dasyphyllum* (Bremer, 1994; Stuessy, Sang & DeVore, 1996; Gustafsson et al., 2001; Urtubey & Stuessy, 2001; Gruenstaeudl et al., 2009) but none of them representative of taxon sampling from each genus. Furthermore, these phylogenetic results proposed conflicting hypotheses for the relationships within the subfamily, especially regarding the monophyly of *Dasyphyllum* and its infrageneric classification.

Therefore, the main purposes of this work were to: (1) infer the intergeneric relationships of *Dasyphyllum* based on three molecular markers (plastid *trn*L-*trn*F and *psb*A-*trn*H, and nuclear ITS) using a broad taxonomic sampling of Barnadesioideae; (2) test the current circumscription of *Dasyphyllum* and its infrageneric classification according to Saavedra (2011) and Saavedra et al. (2018), and update the taxonomy; and (3) investigate the character evolution of *Dasyphyllum*.

## Materials and Methods

### Taxon sampling

A total of 60 out of the 85 species of Barnadesioideae, representing all nine genera, were sampled in this study. This included 27 of the 33 species (82%) from all sections of *Dasyphyllum* (Saavedra, 2011; Saavedra et al., 2018), covering most of its morphological diversity and geographical distribution. The six species missing in our analysis were not included due to unsuccessful DNA extractions or because we could not obtain voucher materials on loan for DNA extraction. A total of 61 accessions were newly sequenced and deposited in GenBank (Table S1); additionally, 125 accessions were obtained from previous studies (Gustafsson et al., 2001; Gruenstaeudl et al., 2009; Katinas et al., 2008; Funk & Roque, 2011; Funk et al., 2014; Table S2). Two species of *Mutisia* (Asteraceae: Mutisioideae) and one species of *Calycera* (Calyceraceae) were used as outgroups. All phylogenetic trees were rooted against to Calyceraceae, the sister family of Asteraceae (Barker et al., 2016; Panero & Crozier, 2016).

### Molecular analysis

Total genomic DNA was extracted from three to five mg of silica-gel dried leaves using the Qiagen DNeasy Plant Mini Kit (Qiagen, Valencia, CA, USA) according to the instructions by the manufacturer. We selected and amplified three regions previously used to infer the phylogenetic relationships in Barnadesioideae: *trn*L-*trn*F using primers “c” and “f” (Taberlet et al., 1991); *psb*A-*trn*H using primers “psbAF” and “trnHR” (Sang, Crawford & Stuessy, 1997); and ITS using primers 18s F and 26s R (Gruenstaeudl et al., 2009). PCR reaction mixtures and purification were carried out after as per Bruniera, Kallunki & Groppo (2015). Thermal cycling for plastid amplification was performed using initial denaturation at 94 °C (8 min), followed by 30 cycles at 94 °C (1 min), 54 °C (1 min), 72 °C, (2 min), ending with an elongation at 72 °C (3 min). Nuclear thermal cycling was performed according to Barfuss et al. (2005), except for the annealing temperature of 62 °C (used in this study). Sequencing of the amplified DNA regions was performed at CREBIO (Jaboticabal, São Paulo, Brazil) with the same primers used for PCR amplification.

Sequences were assembled and edited using the Biological Sequence Alignment Editor (BioEdit), version 7.2.5 (Hall, 1999). We performed sequence alignments using MAFFT version 7 (Katoh & Standley, 2013) with default parameters, followed by manual adjustments with Mesquite version 3.51 (Maddison & Maddison, 2018). All data matrices generated are included in Data S1.

Phylogenetic trees for each molecular region and the combined datasets were constructed under parsimony (PA), maximum likelihood (ML), and Bayesian inference (BI). PA analyses were performed in PAUP* version 4.0b10 (Swofford, 2002). Heuristics searches were performed with 10,000 random addition sequence replicates holding 10 trees at each step, tree-bisection-reconnection (TBR) branch swapping, with the “steepest descent” and “multrees” options off. All characters were unordered and equally weighted. Bootstrapping was implemented with 1,000 pseudoreplicates, 10,000 random taxon addition, and TBR branch-swapping algorithm. Bootstrap (BP) support values in the following ranges were considered strong (>88%), moderate (76–87%), weak (63–75%), and ambiguous (<63%) following Bruniera, Kallunki & Groppo (2015).

Maximum likelihood and BI analyses were performed on the CIPRES Science Gateway (Miller, Pfeiffer & Schwartz, 2010). The most appropriate model of sequence evolution for each matrix was selected using the Akaike information criterion (Akaike, 1973) in jModelTest version 2.1.9 (Posada, 2008; Darriba et al., 2012). Selected models were GTR + I + G for ITS and GTR + G for both *psb*A-*trn*H and *trn*L-*trn*F.

Maximum likelihood analyses were performed using RaxML version 8 (Stamatakis, 2014) associated with a rapid BP analysis of 1,000 replicates under the GTRCAT model. ML BP were interpreted as in the PA analyses.

Bayesian inference analyses were performed in MrBayes version 3.2.6 (Ronquist et al., 2012) using two independent runs, each run with four simultaneous Markov chains (three heated chains and one cold chain) started from random trees. Analyses were run for 20 million generations, and values were sampled every 1,000 generations. The stationarity and convergence of runs, as the effective sample size ≥200 were ascertained using Tracer version 1.6 (Rambaut et al., 2013). The first 25% of the sample trees were discarded as burn-in and a 50% majority-rule consensus tree was calculated from the remaining trees using the sumt option. Posterior probabilities (PP) above 0.95 were considered as strong support.

The incongruence length difference test (ILD; Farris et al., 1995) was performed to test the congruence between the plastid marker datasets (*psb*A-*trn*H and *trn*L-*trn*F) and the combined marker datasets generated in this study (*psb*A-*trn*H, *trn*L-*trn*F, and ITS). The ILD test was performed using PAUP* version 4.0b10 (Swofford, 2002) with 1,000 replicates and the same parameters used for PA searches.

### Taxonomy

The electronic version of this article in portable document format will represent a published work according to the international code of nomenclature for algae, fungi, and plants (ICN), and hence the new names contained in the electronic version are effectively published under that Code from the electronic edition alone. In addition, new names contained in this work which have been issued with identifiers by IPNI will eventually be made available to the global names index. The IPNI LSIDs can be resolved and the associated information viewed through any standard web browser by appending the LSID contained in this publication to the prefix “http://ipni.org/”. The online version of this work is archived and available from the following digital repositories: PeerJ, PubMed Central, and CLOCKSS.

### Ancestral state reconstruction

In order to understand how the morphological features traditionally used to recognize the infrageneric groups have evolved in *Dasyphyllum*, we reconstructed ancestral character traits using the Bayesian majority-rule consensus tree based on the combined datasets (*trn*L-*trn*F, *psb*A-*trn*H, and ITS) and further ultrametrized using the chronopl function with default parameters in the R package “ape” (Paradis, Claude & Strimmer, 2004). Ancestral state reconstructions were estimated from 1,000 iterations of Bayesian stochastic character mapping (Bollback, 2006) using the function make.simmap in the R package phytools (Revell, 2012). Coding of morphological characters was extracted from the literature (Cabrera, 1959; Stuessy, Urtubey & Gruenstaeudl, 2009; Funk & Roque, 2011; Saavedra, 2011; Saavedra, Monge & Guimarães, 2014; Saavedra et al., 2018) and from examination of specimens from the following herbaria: ALCB, B, BAF, BHCB, BM, BOTU, BR, CEN, CEPEC, CESJ, CONC, CVRD, EAC, ESA, GFJP, GOET, GUYN, HB, HEPH, HPBR, HRCB, HST, HUEFS, HUFU, IBGE, ICN, IPA, JBP, K, LP, M, MBM, MBML, MO, MOSS, NY, OUPR, P, PACA, PEUFR, QCA, R, RB, S, SI, SP, SPF, SPFR, UB, UEC, UFG, UFMT, UFP, UFRN, UPCB, US, VIC (herbaria acronyms follow Thiers, 2018). A list of morphological characters and their character state coding used for the ancestral state reconstruction is detailed in Table 1.

**Table 1:** Diagnostic feature coding used to infer the Bayesian stochastic character mapping analyses.

Taxon	Leaf venation	Anther apical appendage	Involucre size	Capitula arrangement
*Arnaldoa macbrideana*	0	0	0	0
*Arnaldoa weberbaueri*	0	0	0	0
*Dasyphyllum argenteum*	0	1	1	1
*Dasyphyllum armatum*	0	1	1	1
*Dasyphyllum brasiliense*	0	1	1	1
*Dasyphyllum brevispinum*	0	1	1	1
*Dasyphyllum colombianum*	0	1	1	1
*Dasyphyllum diacanthoides*	1	2	1	0
*Dasyphyllum diamantinense*	0	1	1	1
*Dasyphyllum donianum*	0	1	0	0
*Dasyphyllum excelsum*	1	2	1	1
*Dasyphyllum ferox*	0	1	1	1
*Dasyphyllum flagellare*	0	1	1	1
*Dasyphyllum floribundum*	0	1	1	1
*Dasyphyllum fodinarum*	0	1	0	0
*Dasyphyllum hystrix*	0	1	1	0
*Dasyphyllum inerme*	0	1	1	1
*Dasyphyllum lanceolatum*	0	1	1	1
*Dasyphyllum leptacanthum*	0	1	1	0
*Dasyphyllum popayanense*	0	1	1	1
*Dasyphyllum reticulatum*	0	1	0	0
*Dasyphyllum spinescens*	0	1	1	1
*Dasyphyllum sprengelianum*	0	1	0	0
*Dasyphyllum trichophyllum*	0	1	0	0
*Dasyphyllum vagans*	0	1	1	1
*Dasyphyllum sp. nov*. (1)	0	1	0	0
*Dasyphyllum sp. nov*. (2)	0	1	1	1
*Dasyphyllum sp. nov*. (3)	0	1	1	1
*Dasyphyllum sp. nov*. (4)	0	1	1	1
*Fulcaldea laurifolia*	0	0	1	1
*Fulcaldea stuessy*	0	0	1	1

**Note:**

Leaf venation: (0) Acrodomous, (1) Pinnate. Anther apical appendage: (0) Acute, (1) Bifid, (2) Obtuse. Involucre size: (0) ≥20 mm, (1) ≤18 mm. Capitula arrangement: (0) Solitary or few capitula (1) Capitula arranged in synflorescences (corymbiform cymes).

Scanning electron microscopy was used to examine anther apical appendages in two species of *Dasyphyllum*. Dried florets were rehydrated with hot water and stored in 70% ethanol; then, anthers were critically point dried, sputter coated with gold and analyzed using an EVO 50 scanning electron microscope (Carl Zeiss, Cambridge, UK).

## Results

### Phylogenetic analyses

**Figure 2:**
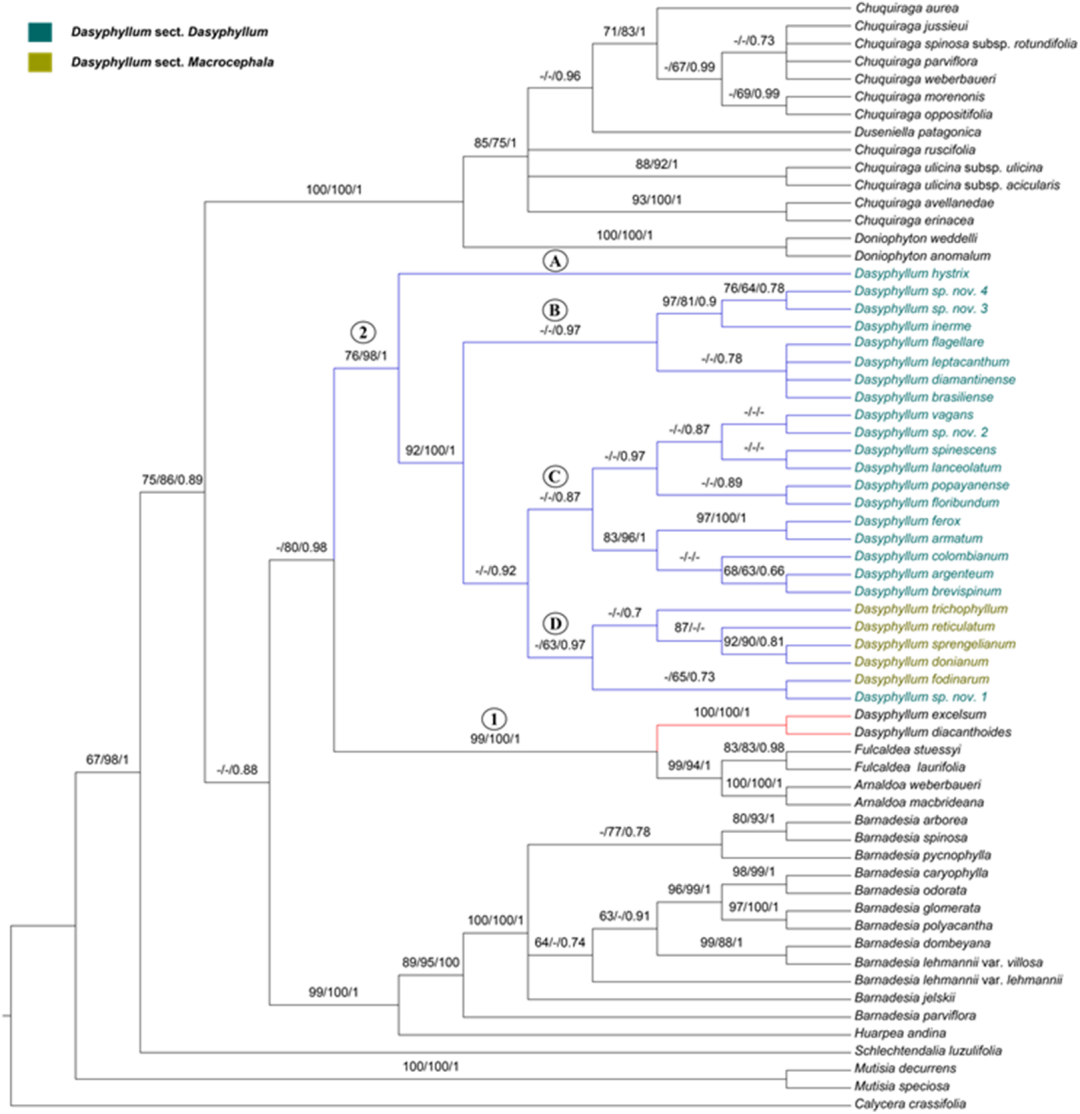
Phylogenetic relationships of *Dasyphyllum* based on combined datasets inferred from Bayesian inference. Support values are indicated above the branches in the order of parsimony, maximum likelihood, and Bayesian analyses. Support values less than 63% are indicated by a dash (–). Capital letters on internal clades of *Dasyphyllum* are discussed in the article.

The ILD test did not indicate incongruences between the plastid and combined datasets (*P* > 0.05), thus allowing both to be used for further phylogenetic analyses. Moreover, based on the results of BP and PP (>80), we did not find any evidence of significant incongruence among the relationships that differed between the trees (Fig. 2; Figs. S1–S4). Therefore, we decided to discuss our results based on the combined analysis of the three regions as it includes the largest number of taxa (Fig. 2). Our combined alignment consisted of 2,414 bp (*trn*l-*trn*F = 912 bp; *psb*A-*trn*H = 537; ITS = 965 bp) for 63 taxa (see summary statistics for each dataset in Table 2).

**Table 2:** Summary statistics of the datasets used in this study.

	*trnL-trnF*	*psbA-trnH*	ITS	Plastid dataset	Combined dataset
Number of taxa included	53	49	60	53	63
Aligned length (BP)	912	537	965	1,449	2,414
Number of constant characters (%)	807 (88.49)	386 (71.88)	499 (51.71)	1,139 (78.61)	1,692 (70.09)
Number of variable characters (%)	105 (11.51)	151 (28.12)	466 (48.29)	310 (21.39)	722 (29.91)
Number of parsimony informative characters (%)	53 (5.81)	61 (11.36)	346 (35.85)	114 (7.87)	460 (19.06)
Tree length of best parsimony tree (steps)	120	222	1,375	348	1,743
Number of most parsimonious trees	20.251	3.120	309	11.337	3,475
Consistency index (CI)	0.9083	0.8018	0.4611	0.1753	0.4102
Retention index (RI)	0.9722	0.8739	0.4412	0.9181	0.8314

In all phylogenetic hypotheses, *Dasyphyllum* was found to be non-monophyletic due to the highly supported position of *D. diacanthoides* and *D. excelsum* (formely subgenus *Archidasyphyllum*) as sister clade to *Fulcaldea* and *Arnaldoa* (Fig. 2, Node 1, PA BP 99%, ML BP 100%, PP 1).

*Dasyphyllum sensu stricto*, defined here by excluding *D. diacanthoides* and *D. excelsum,* was recovered as monophyletic with moderate or strong support (Fig. 2; Node 2; PA BP 76%, ML BP 98%, PP 1). However, at the intrageneric level, both currently-accepted sections (*Dasyphyllum* and *Macrocephala*) were found to be non-monophyletic. Members of *Dasyphyllum sensu stricto* are divided into four main lineages: (1) lineage “A” is composed only of *D. hystrix* and is sister to the rest of the genus (PA BP 76%, ML BP 98%, PP 1); (2) lineage “B” comprises seven species classified in section. *Dasyphyllum* of Saavedra (2011) and is only supported in the Bayesian analysis (PP 0.97); (3) lineage “C” is composed of 11 species, including approximately 46% of the species currently classified in sect. *Dasyphyllum* of Saavedra (2011), with no strong support in any analysis; (4) lineage “D” is composed of five of the six species positioned in sect. *Macrocephala* of Saavedra et al. (2018), plus one undescribed Brazilian species (*Dasyphyllum* sp. nov. 1) previously positioned in sect. *Dasyphyllum* of Saavedra (2011), and it is only strongly supported in the Bayesian analysis (PP 0.97).

The phylogenetic analyses of individual (Figs. S1 and S2) and combined (Fig. S3) plastid marker datasets do not have good resolutions or supports and do not clarify the relationships of *Dasyphyllum sensu stricto* and the rest of the subfamily. On the other hand, in the ITS (Fig. S4) and combined phylogenies (Fig. 2), *Dasyphyllum* is placed as sister to the clade comprising *Arnaldoa*, *Fulcaldea, D. diacanthoides*, and *D. excelsum* ((PA BP 98%, ML BP 100%, PP1) support values for ITS; PA BP 99%, ML BP 100%, PP 1 support values for combined).

### Ancestral state reconstruction analyses

**Figure 3:**
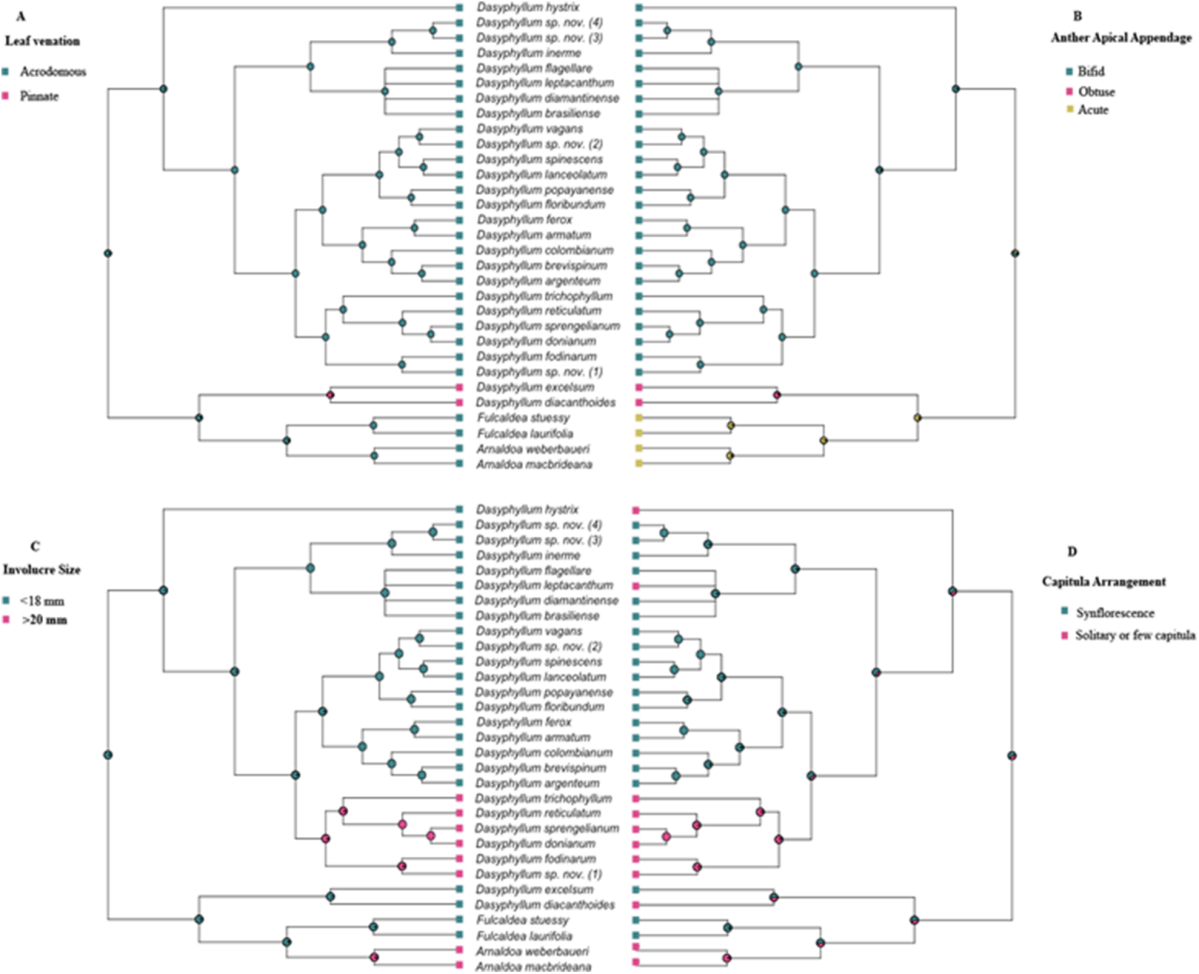
History of the morphological characters traditionally used to circumscribe infrageneric groups of *Dasyphyllum*. (A) Leaf venation. (B) Anther apical appendage. (C) Involucre size. (D) Capitula arrangement. Squares to the right and left of the phylogeny are color-coded according to each character state. Pie charts at nodes represent posterior probabilities of ancestral states using Bayesian inference.

Bayesian stochastic character mapping demonstrated that the ancestral condition in *Dasyphyllum sensu stricto* is acrodromous leaf venation (PP = 0.99; Fig. 3A), bifid anther apical appendages (PP = 0.96; Fig. 3B), and small involucres (PP = 0.99; Fig. 3C) with capitula arranged into an synflorescence (PP = 0.66; Fig. 3D). Pinnate venation (Fig. 3A) and obtuse anther apical appendages (Fig. 3B) evolved in the ancestor of the clade comprising *D. diacanthoides* and *D. excelsum* (PP 0.95 and PP 0.82, respectively). The larger involucre larger (≥20 mm) is inferred to have evolved twice, since it appears in the ancestor of lineage “D” (PP 0.98), and in the *Arnaldoa* clade (PP 0.95). Regarding capitula arrangement, solitary, or arranged in few inflorescences (2–4) is a derived state and appears at least five times over the evolutionary history of the group.

## Discussion

Previous molecular phylogenetic hypotheses aimed to clarify the intergeneric relationships within Barnadesioideae, but they only included a limited taxonomic sampling from each genus (Gustafsson et al., 2001; Gruenstaeudl et al., 2009). Our combined phylogeny greatly improves the taxonomic coverage by including almost 82% of the species recognized as belonging to *Dasyphyllum*. The results obtained here allowed us to review the generic taxonomy and to discuss the morphological features used to recognize the infrageneric groups within this genus.

### Re-circumscription of *Dasyphyllum*

**Figure 4:**
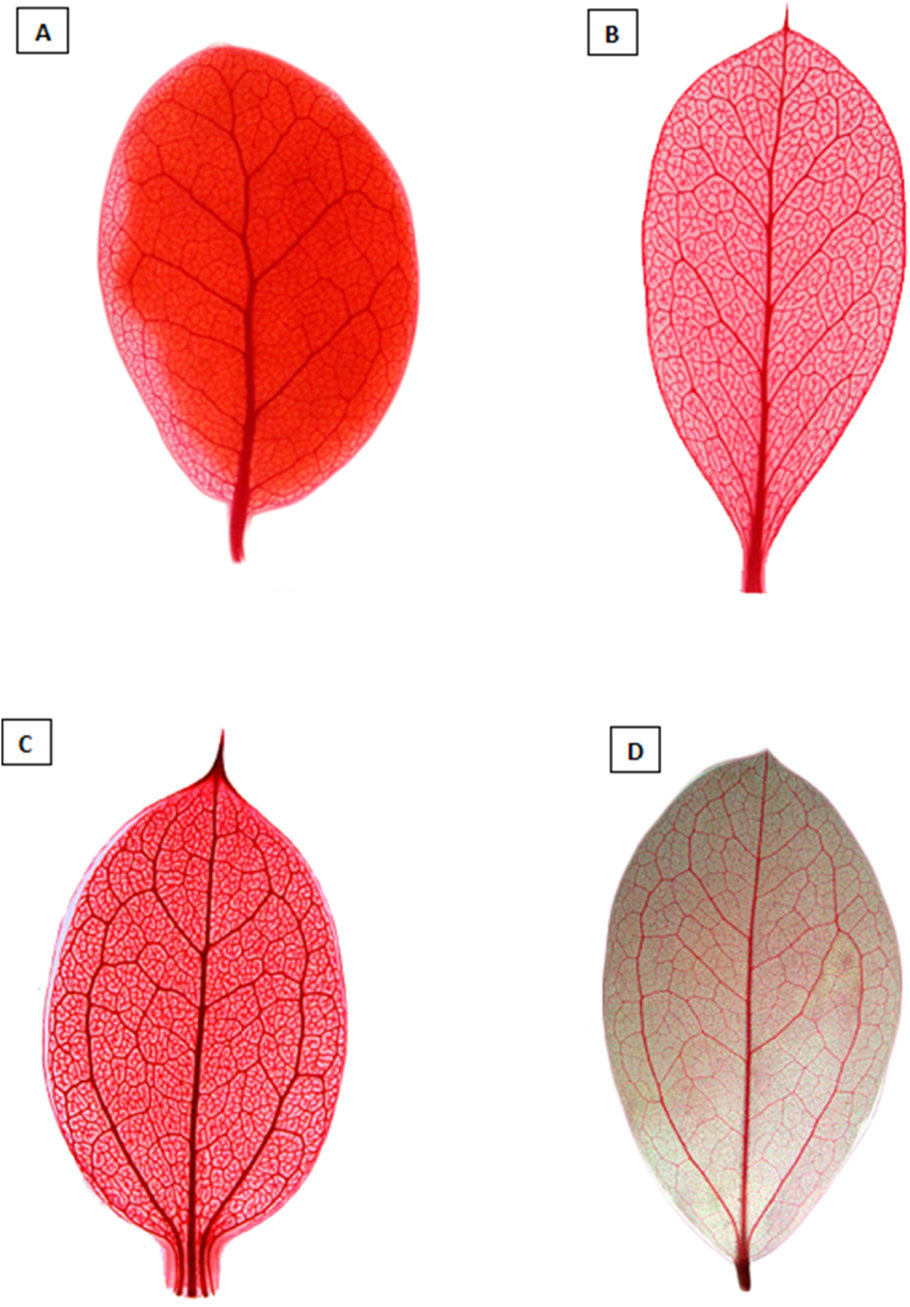
Diaphanized leaves showing the differences in venation. (A and B) show the pinnate venation of *Dasyphyllum* subgenus *Archidasyphyllum*. (C and D) show the acrodomous venation of *Dasyphyllum* sensu stricto. Photos: (A) *Dasyphyllum excelsum*. (B) *Dasyphyllum diacanthoides*. (C) *Dasyphyllum argenteum*. (D) *Dasyphyllum brasiliense*. All photographs were extracted from Saavedra (2011).

**Figure 5:**
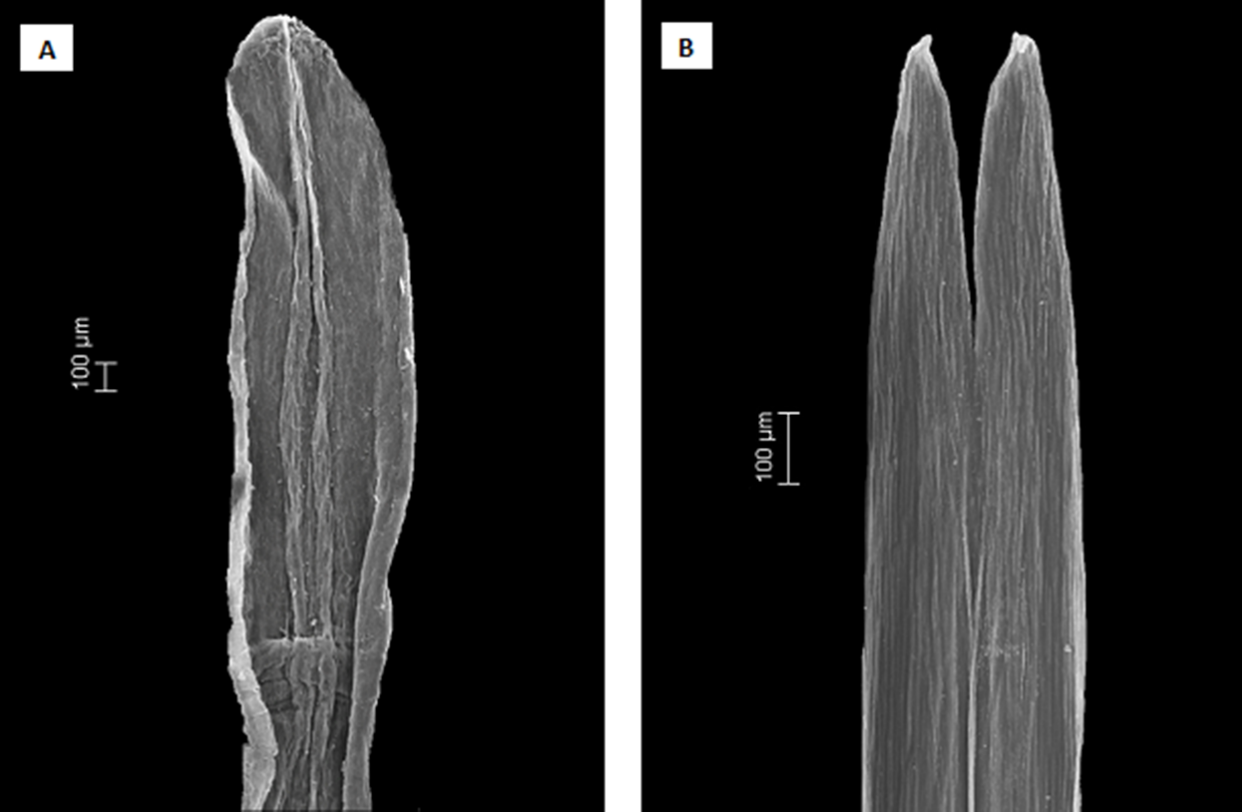
Scanning electron microscopy images showing the differences in anther apical appendages. (A) apical appendages obtuse of *Dasyphyllum diacanthoides* (*Dasyphyllum* subgenus *Archidasyphyllum*). (B) apical appendages bifid of *Dasyphyllum trichophyllum* (Baker) Cabrera (*Dasyphyllum* sensu stricto).

All phylogenetic analyses show that, as traditionally circumscribed, *Dasyphyllum* is non-monophyletic due to the well-supported placement of *D. diacanthoides* and *D. excelsum*, which belong to *Dasyphyllum* subg. *Archidasyphyllum*, sensu Cabrera (1959), in a clade sister to *Arnaldoa* and *Fulcaldea* (Fig. 2; Figs. S1–S4), a finding that confirms previous studies based on molecular data (Gustafsson et al., 2001; Gruenstaeudl et al., 2009; Funk & Roque, 2011; Padin, Calviño & Ezcurra, 2015). Despite their shared Andean distribution, the clade comprising *Arnaldoa*, *Fulcaldea*, *D. diacanthoides*, and *D. excelsum* is morphologically diverse and well-defined into distinct genera: *Fulcaldea* comprises two species of shrubs or small trees found in southern Ecuador, northern Peru, and Brazil; the species of this genus are distinguished by having single-flowered capitula, a style with subapical swelling, and villose pappus with red or pink bristles (Gustafsson et al., 2001; Stuessy, Urtubey & Gruenstaeudl, 2009; Funk & Roque, 2011). On the other hand, *Arnaldoa* comprises three shrubs species distributed in Ecuador and northern Peru; they are distinguished by their large and solitary capitula with sub-bilabiate, white, orange, or purple corollas (Stuessy & Sagástegui, 1993; Ulloa, Jørgensen & Dillon, 2002). In contrast, *D. diacanthoides* and *D. excelsum* are restricted to the relict *Nothofagus* forests of central Chile and adjacent areas of Argentina (Cabrera, 1959; Gustafsson et al., 2001; Gruenstaeudl et al., 2009; Stuessy, Urtubey & Gruenstaeudl, 2009) and are easily distinguished from *Fulcaldea* and *Arnaldoa* because *D. diacanthoides* and *D. excelsum* are tall trees (up to 30 m) with leaves showing pinnate venation (Figs. 3A, 4A and 4B), solitary or spiciform (Fig. 3D), gynodioecious or monoecious capitula with more than one flower, and emarginated or obtuse anther apical appendages (Figs. 3B and 5A; Cabrera, 1959; Saavedra, 2011). Due to the great morphological diversity, classifying *Arnaldoa*, *Fulcaldea*, and *Dasyphyllum* subg. *Archidasyphyllum* together in one single unit would result in several undesirable taxonomic changes and create a drastically broader genus concept with no obvious morphological support.

Instead, we propose a new circumscription of *Dasyphyllum* by elevating subg. *Archidasyphyllum* to the generic rank, *Archidasyphyllum*. This proposal is phylogenetically well-supported and consistent with leaf venation pattern (Fig. 4), anther apical appendage shape (Fig. 5), and distributional data (Stuessy, Sang & DeVore, 1996; Gruenstaeudl et al., 2009; Saavedra, 2011). New combinations and a key for this genus, as well as other commentaries about the distribution and phenology of the species, are presented at the end of the manuscript.

### *Dasyphyllum sensu stricto*—intergeneric relationships and infrageneric classification

The phylogenetic relationships of *Dasyphyllum* with genera in Barnadesioideae remains unresolved. Our phylogenetic hypotheses are consistent with the placement of *Dasyphyllum* as a sister clade to the clade comprising *Arnaldoa*, *Fulcaldea*, and *Archidasyphyllum* (Fig. 2; Fig. S4). This relationship was also supported by previous molecular phylogenetic analyses (Gustafsson et al., 2001; Gruenstaeudl et al., 2009; Funk & Roque, 2011).

As stated in the introduction, *Dasyphyllum sensu stricto* (*D*. subgenus *Dasyphyllum*, sensu Cabrera, 1959) has been traditionally divided into two sections based on involucre size and capitula arrangement. Our results indicated that neither section is monophyletic (Fig. 2). Section *Macrocephala* comprises six species found in adjacent areas of Bolivia and Paraguay (Saavedra et al., 2018) that share the presence of few large capitula, solitary or in small groups of heads (Figs. 1A and 1B), and it can be recognized as a monophyletic group by inclusion of *Dasyphyllum*. sp. nov. (1). Although these morphological features have evolved more than once over evolutionary history (Figs. 3C and 3D), they are useful to define this clade. Moreover, our Bayesian stochastic mapping analyses showed that the character states previously used to define section *Dasyphyllum* (involucre up to 18 mm in length and capitula arranged in synflorescences; Figs. 3C and 3D) are plesiomorphic, and therefore cannot be used to delimitate infrageneric groups as previously proposed by Cabrera (1959) and Saavedra (2011).

Based on our taxonomic sampling, species of *Dasyphyllum sensu stricto* fall into four heterogeneous and poorly supported lineages (Fig. 2; lineages A–D). Therefore, the results of this work do not corroborate the subdivision of *Dasyphyllum* into sections and they should be abandoned.

### Taxonomic treatment

***Archidasyphyllum*** (Cabrera) P.L.Ferreira, Saavedra & Groppo, **stat. nov.** ≡ *Dasyphyllum* subgenus *Archidasyphyllum* Cabrera, *Revista Mus. de La Plata*, *Secc. Bot*., 9(38): 44. 1959. Type: *Archidasyphyllum diacanthoides* (Less.) P.L.Ferreira, Saavedra & Groppo.

*Etymology. Archi* (Greek) = First, Primitive; *Dasyphyllum* = genus that belongs to Barnadesioideae. Cabrera (1959) suggested that *Dasyphyllum* subgenus *Archidasyphyllum* is the earliest diverging group of the subfamily Barnadesioideae.

Key to species of *Archidasyphyllum*

1. Capitula solitary on the branches*A. diacanthoides*
1. Capitula arranged in spiciform synflorescences*A. excelsum*

New combinations:

***Archidasyphyllum diacanthoides*** (Less.) P.L.Ferreira, Saavedra & Groppo **comb. nov.** ≡ *Flotovia diacanthoides* Less, *Syn. Gen. Compos*.: 95. 1832. ≡ *Piptocarpha diacanthoides* (Less.) Hook. & Arn., Comp. Bot. Mag. 1: 110. 1835. ≡ *Dasyphyllum diacanthoides* (Less.) Cabrera, *Revista Mus. La Plata, Secc. Bot*., 9(38): 44. 1959. - Type: Chile, Antuco, *E.F. Poeppig [Coll. pl. Chil. III, Syn. pl. Amer. austr. msc., Diar. 793]*, XII.1828 (*Lectotypus hic designatus*: P! [P00703408]; *Isolectotypi*: B † [photo F! [F0BN015834]], BM! [BM001010220], BR! [BR541864], M! [M-0030607], NY! [00169364, 00169365]).

*Distribution and Habitat—Archidasyphyllum diacanthoides* is distributed in southern Chile and adjacent areas of Argentina between 38° and 43°S. This species is found in forested areas ranging from 400 to 1,200 m in elevation.

*Phenology*—Flowering from November to April.

*Note—Flotovia diacanthoides* was described by Lessing (1832) based on the material “*Chuquiraga leucoxilon* Pöpp. mss. n. 793” (*nomen nudum*) collected by Poeppig. According to Stafleu (1969), the plants collected by Poeppig in Chile were distributed by Kunze under the designation “Coll. pl. Chi.”. Although all the type materials assigned to *Flotovia diacanthoides* are indicated with the phrase “Coll. pl. Chl.”, we designated the sheet deposited at P herbarium as the lectotype because it is the only material which also bears a handwritten label “N. 793 *Chuquiraga leucoxilon*”.

***Archidasyphyllum excelsum*** (D. Don) P.L.Ferreira, Saavedra & Groppo **comb. nov.** ≡ *Chuquiraga excelsa* D. Don, Phil. Mag. 11: 392. 1832. ≡ *Piptocarpha excelsa* (D. Don) Hook. & Arn., Comp. Bot. Mag. 1:110. 1835. ≡ *Dasyphyllum excelsum* (D. Don) Cabrera, *Revista Mus. La Plata, Secc. Bot.,* 9(38): 46. 1959. *Typus:* Chile, Valparaiso, *H. Cuming 328*, 1832 (*Lectotypus hic designatus*: K! [K000527920]; *Isolectotypi*: BM! [000522369], FI [107436 [image!]], GH [00006351 [image!]], P! [P00703407]).

*Distribution and Habitat—Archidasyphyllum excelsum* is endemic to central Chile between 32° and 34°S. This species is found in forested areas ranging from 350 to 900 m in elevation.

*Phenology*—Flowering from November to April.

*Note*—According to Stafleu & Cowan (1976–1998), the herbarium of David Don was donated to the Linnean Society of London and should be conserved at the LINN herbarium. However, we have been unable to trace this material and we designated the lectotype in the K herbarium due to the specimen being well-represented in its reproductive and vegetative forms, besides the high preservation of the material.

## Conclusions

This study comprises the most extensive molecular sampling for *Dasyphyllum* to date and provides a sound foundation for the re-circumscription of the genus. In so doing, it also sheds new light on the evolution of morphological features. Our phylogenetic analysis demonstrated that as currently circumscribed, *Dasyphyllum* is not monophyletic, because of *D. diacanthoides* and *D. excelsum* (*Dasyphyllum* subgenus *Archidasyphyllum*) being placed outside the genus, as sister to a clade comprising *Arnaldoa* and *Fulcaldea*. A well-supported phylogeny coupled with morphological and biogeographical data corroborate our taxonomic decision to elevate *Dasyphyllum* subgenus *Archidasyphyllum* to generic status as *Archidasyphyllum*. In addition, both sections of *D. sensu stricto* were also rejected. However, we prefer not to propose a new infrageneric classification until new data with unequivocal synapomorphies for the internal clades are available. Moreover, phylogenetic relationships between *Dasyphyllum* and other genera of Barnadesioideae remain to some extent unresolved. We suggest that future studies including additional characters from phylogenomics might better clarify the relationships of the internal clades in *Dasyphyllum*, as well as the relationships within the whole subfamily Barnadesioideae.

## Supplemental Information

10.7717/peerj.6475/supp-1 Supplemental Information 1 Fig. S1. Phylogenetic relationships of Dasyphyllum based on trnl-trnF marker inferred from Bayesian inference. Support values are indicated above the branches in the order of parsimony, maximum likelihood, and Bayesian analyses. Support values lower than 63% are indicated by a dash (–).

10.7717/peerj.6475/supp-2 Supplemental Information 2 Fig. S2. Phylogenetic relationships of Dasyphyllum based on psbA-trnH marker inferred from Bayesian inference. Support values are indicated above the branches in the order of parsimony, maximum likelihood, and Bayesian analyses. Support values lower than 63% are indicated by a dash (–).

10.7717/peerj.6475/supp-3 Supplemental Information 3 Fig. S3. Phylogenetic relationships of Dasyphyllum based on the plastid markers (psbA-trnH and trnl-trnF) inferred from Bayesian inference. Support values are indicated above the branches in the order of parsimony, maximum likelihood, and Bayesian analyses. Support values lower than 63% are indicated by a dash (–).

10.7717/peerj.6475/supp-4 Supplemental Information 4 Fig. S4. Phylogenetic relationships of Dasyphyllum based on the ITS marker inferred from Bayesian inference. Support values are indicated above the branches in the order of parsimony, maximum likelihood, and Bayesian analyses. Support values lower than 63% are indicated by a dash (–).

10.7717/peerj.6475/supp-5 Supplemental Information 5 Table S1. List of taxa sampled, voucher specimens (herbarium acronym) and GenBank accession numbers generated in this study.

10.7717/peerj.6475/supp-6 Supplemental Information 6 Table S2. List of taxa sampled and Genbank accession numbers extracted from previous studies.

10.7717/peerj.6475/supp-7 Supplemental Information 7 Molecular datasets.

## Additional Information and Declarations

### Data Availability

The following information was supplied regarding data availability: The GenBank accession numbers are provided in Table S1 and Table S2. Molecular matrices are provided in the Supplemental Data S1.

### New Species Registration

The following information was supplied regarding the registration of a newly described species: *Archidasyphyllum* (Cabrera) P.L. Ferreira, Saavedra & Groppo LSID: 77194153-1. *Archidasyphyllum diacanthoides* (Less.) P.L. Ferreira, Saavedra & Groppo LSID: 77194155-1. *Archidasyphyllum excelsum* (D. Don) P.L. Ferreira, Saavedra & Groppo LSID: 77194156-1.
