# Supplementary material for: Phylogeny and circumscription of Dasyphyllum (Asteraceae: Barnadesioideae) based on molecular data with the recognition of a new genus, Archidasyphyllum
Source: PeerJ. 2019 Feb 27;7:e6475. doi: 10.7717/peerj.6475 (PMC6397630; doi:10.7717/peerj.6475)
Supplement: Supplemental Information 3 — Support values are indicated above the branches in the order of parsimony, maximum likelihood, and Bayesian analyses. Support values lower than 63% are indicated by a dash (–). [file peerj-07-6475-s003.pdf]

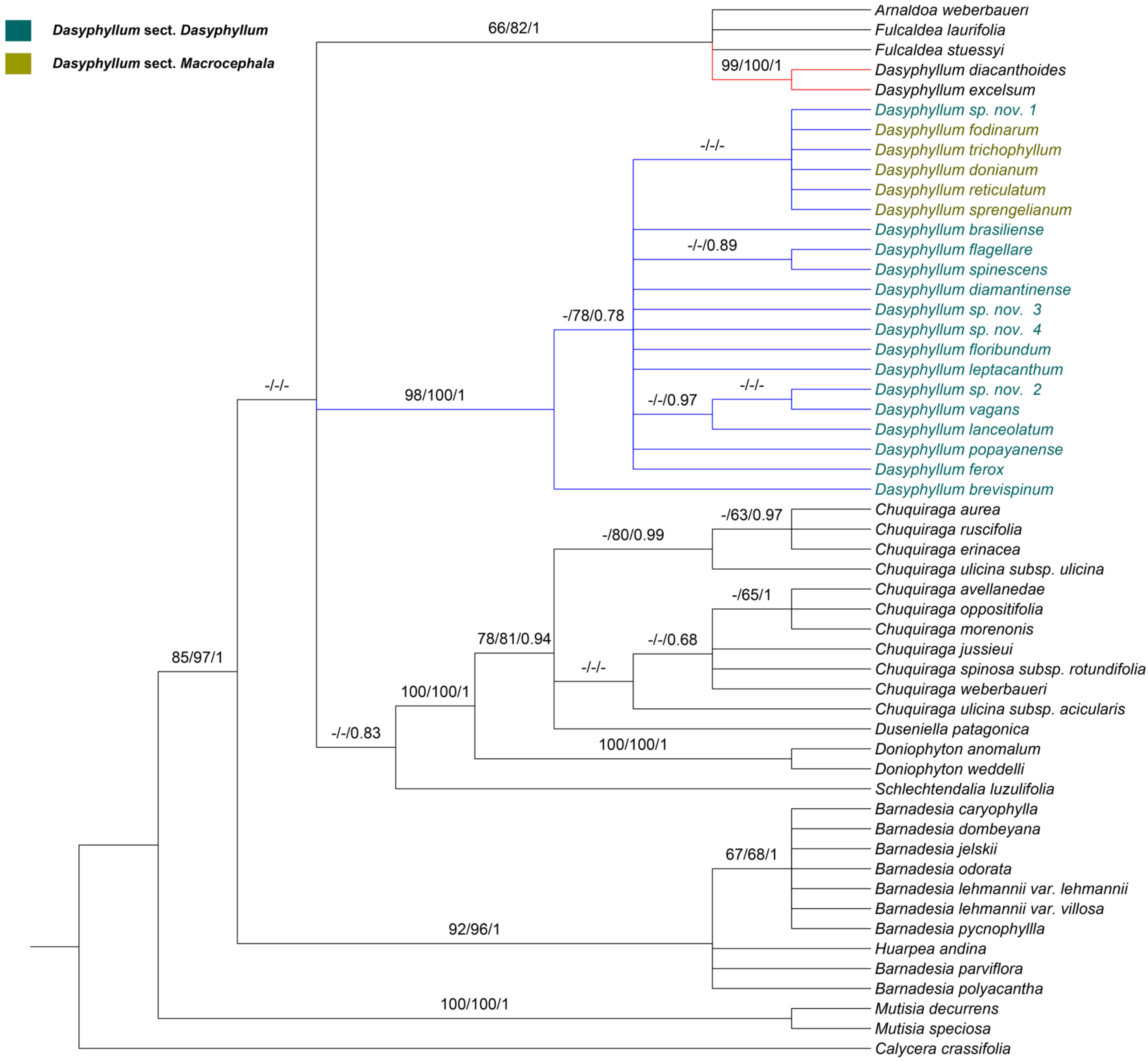

Phylogenetic relationships of *Dasyphyllum* based on the plastid markers (*psb*A-*trn*H and *trn*l-*trn*F) inferred from Bayesian inference. Support values are indicated above the branches in the order of parsimony, maximum likelihood, and Bayesian analyses. Support values lower than 63% are indicated by a dash (–).
