# Supplementary material for: Phylogeny and circumscription of Dasyphyllum (Asteraceae: Barnadesioideae) based on molecular data with the recognition of a new genus, Archidasyphyllum
Source: PeerJ. 2019 Feb 27;7:e6475. doi: 10.7717/peerj.6475 (PMC6397630; doi:10.7717/peerj.6475)
Supplement: Supplemental Information 4 — Support values are indicated above the branches in the order of parsimony, maximum likelihood, and Bayesian analyses. Support values lower than 63% are indicated by a dash (–). [file peerj-07-6475-s004.pdf]

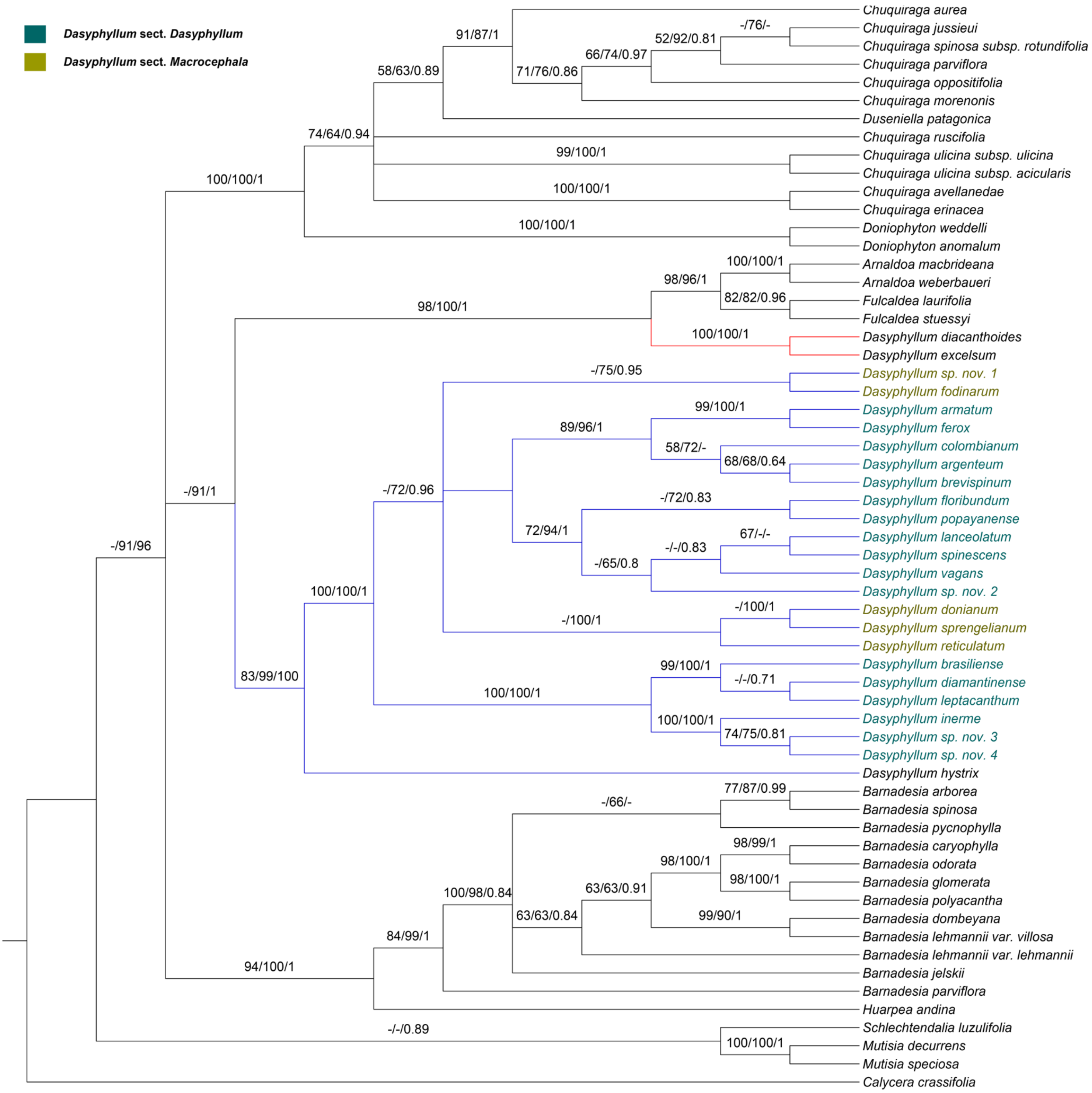

Phylogenetic relationships of *Dasyphyllum* based on the ITS marker inferred from Bayesian inference. Support values are indicated above the branches in the order of parsimony, maximum likelihood, and Bayesian analyses. Support values lower than 63% are indicated by a dash (–).
