# Supplementary material for: Phylogeny and circumscription of Dasyphyllum (Asteraceae: Barnadesioideae) based on molecular data with the recognition of a new genus, Archidasyphyllum
Source: PeerJ. 2019 Feb 27;7:e6475. doi: 10.7717/peerj.6475 (PMC6397630; doi:10.7717/peerj.6475)
Supplement: Supplemental Information 5 [file peerj-07-6475-s005.docx]

**Table S1**. List of taxa sampled, voucher specimens (herbarium acronym) and GenBank accession numbers generated in this study.

| Taxon | Voucher | *trnL-trnF*  (intron plus intergenic spacer) | *psbA*-*trnH* | ITS |
| --- | --- | --- | --- | --- |
| *Barnadesia caryophylla* (Vell.) S.F. Blake | P.L. Ferreira *et al.* 7 (SPFR) | MK034077 | MK034055 | MK034038 |
| *Dasyphyllum brasiliense* (Spreng.) Cabrera | M.M. Saavedra *et al.* 521 (RB) | MK034078 | MK034056 | MK034039 |
| *Dasyphyllum diacanthoides* (Less.) Cabrera | M. Monge *et al.* 2073 (SPFR) | MK034079 | MK034057 |  |
| *Dasyphyllum diamantinense* Saavedra & M. Monge | P.L. Ferreira *et al.* 8 (SPFR) | MK034080 | MK034058 | MK034041 |
| *Dasyphyllum donianum* (Gardner) Cabrera | M.M. Saavedra *et al*. 993 (RB) | MK034081 | MK034059 | MK034042 |
| *Dasyphyllum excelsum* (D. Don) Cabrera | C. Luz et. al. 195 (SPFR) | MK034082 | MK034060 |  |
| *Dasyphyllum flagellare* (Casar.) Cabrera | M.M. Saavedra *et al.* 796 (RB) | MK034083 | MK034061 |  |
| *Dasyphyllum floribundum* (Gardner) Cabrera | M.M. Saavedra *et al.* 997 (RB) | MK034084 | MK034062 | MK034043 |
| *Dasyphyllum fodinarum* (Gardner) Cabrera | C.N. Fraga *et al.* 3330 (RB) | MK034085 | MK034063 | MK034044 |
| *Dasyphyllum hystrix* (Wedd.) Cabrera | J.R. Wood *et al.* 14663 (SPF) |  |  | MK034046 |
| *Dasyphyllum lanceolatum* (Less.) Cabrera | M.M. Saavedra *et al*. 934 (RB) | MK034086 | MK034064 | MK034047 |
| *Dasyphyllum leptacanthum* (Gardner) Cabrera | P.L. Ferreira *et al.* 1 (SPFR) | MK034087 | MK034065 | MK034048 |
| *Dasyphyllum reticulatum* (DC.) Cabrera | C.N. Fraga & N.F.O Mota 3352 (RB) | MK034088 | MK034066 | MK034051 |
| *Dasyphyllum spinescens* (Less.) Cabrera | M.M. Saavedra *et al.* 1018 (RB) | MK034089 | MK034067 | MK034052 |
| *Dasyphyllum sprengelianum* (Gardner) Cabrera | A.P. Fontana *et al.* 6261 (RB) | MK034090 | MK034068 | MK034053 |
| *Dasyphyllum trichophyllum* (Baker) Cabrera | M.M. Saavedra *et al.* 578 (RB) | MK034091 | MK034069 |  |
| *Dasyphyllum vagans* (Gardner) Cabrera | M.M. Saavedra & C.N. Fraga 1039 (RB) | MK034092 | MK034070 |  |
| *Schlechtendalia luzulifolia* Less. | G. Heiden *et al.* 2008 (SPF) | MK034093 | MK034071 |  |
| *Mutisia speciosa* Aiton ex. Hook. | P.L. Ferreira *et al.* 24 (SPFR) | MK034094 | MK034072 | MK034054 |
| *Dasyphyllum sp. nov.* 1 | M.M. Saavedra & L.F. Magnago 1035 (RB) | MK034095 | MK034073 | MK034040 |
| *Dasyphyllum sp. nov.* 2 | M.M. Saavedra *et al.* 1005 (RB) | MK034096 | MK034074 | MK034045 |
| *Dasyphyllum sp. nov.* 3 | M.M. Saavedra *et al.* 508 (RB) | MK034097 | MK034075 | MK034049 |
| *Dasyphyllum sp. nov.* 4 | M.M. Saavedra *et al.* 828 (RB) |  | MK034076 |  |
| *Dasyphyllum sp. nov.* 4 | L. Kollmann 11062 (RB) | MK034098 |  | MK034050 |
