# Supplementary material for: Phylogeny and circumscription of Dasyphyllum (Asteraceae: Barnadesioideae) based on molecular data with the recognition of a new genus, Archidasyphyllum
Source: PeerJ. 2019 Feb 27;7:e6475. doi: 10.7717/peerj.6475 (PMC6397630; doi:10.7717/peerj.6475)
Supplement: Supplemental Information 6 [file peerj-07-6475-s006.docx]

**Table S2.** List of taxa sampled and Genbank accession numbers extracted from previous studies.

| Taxon | *trnL* intron | *trnL – trnF* (intergenic spacer) | *trnL – trnF*  (intron plus intergenic spacer) | *psbA-trnH* | ITS |
| --- | --- | --- | --- | --- | --- |
| *Arnaldoa macbrideana* Ferreyra |  |  |  |  | AF412837 |
| *Arnaldoa weberbaueri* (Muschl.) Ferreyra | EU841055 | EU547617 |  | EU841268 | EU841139 |
| *Barnadesia arborea* Kunth |  |  |  |  | AF412883 |
| *Barnadesia dombeyana* Less. | EU841057 | EU547619 |  | EU841270 | EU841140 |
| *Barnadesia glomerata* Kuntze |  |  |  |  | AF412880 |
| *Barnadesia jelskii* Hieron. | EU841060 | EU547622 |  | EU841273 | EU841143 |
| *Barnadesia lehmannii* Hieron. var. *lehmannii* | EU841058 | EU547620 |  | EU841271 | EU841141 |
| *Barnadesia lehmannii* Hieron. var. *villosa* (I.C. Chung) Urtubey | EU841059 | EU547621 |  | EU841272 | EU841142 |
| *Barnadesia odorata* Griseb. | EU841061 | EU547623 |  | EU841274 | EU841144 |
| *Barnadesia parviflora* Spruce ex Benth. & Hook. f. | AF412925 |  |  |  | AF412876 |
| *Barnadesia polyacantha* Wedd. | AF412924 |  |  |  | AF412886 |
| *Barnadesia pycnophyllla* Muschl. | AF412913 |  |  |  | AF412882 |
| *Barnadesia spinosa* L.f. |  |  |  |  | AF412884 |
| *Chuquiraga aurea* Skottsb. | EU841064 | EU547632 |  | EU841277 | EU841147 |
| *Chuquiraga avellanedae* Lorentz | EU841070 | EU547638 |  | EU841281 | EU841152 |
| *Chuquiraga erinacea* D. Don | EU841065 | EU547633 |  | EU841278 | EU841148 |
| *Chuquiraga jussieui* J.F. Gmel | EU841071 | EU547639 |  | EU841286 | EU841153 |
| *Chuquiraga morenonis* (Kuntze) C. Ezcurra | EU841072 | EU547640 |  | EU841282 | EU841154 |
| *Chuquiraga oppositifolia* D. Don | EU841069 | EU547637 |  | EU841280 | EU841151 |
| *Chuquiraga parviflora* (Griseb.) Hieron. |  |  |  |  | AF412863 |
| *Chuquiraga ruscifolia* D. Don | EU841066 | EU547634 |  | EU841279 | EU841149 |
| *Chuquiraga spinosa* D. Don subsp. *rotundifolia*  (Wedd.) C. Ezcurra | EU841068 | EU547636 |  | EU841285 | EU841150 |
| *Chuquiraga ulicina* Hook. and Arn. subsp. *acicularis* (D. Don) C. Ezcurra | EU841062 | EU547630 |  | EU841275 | EU841145 |
| *Chuquiraga ulicina* Hook. and Arn. subsp. *ulicina* | EU841063 | EU547631 |  | EU841276 | EU841146 |
| *Chuquiraga weberbaueri* Tovar | EU841067 | EU547635 |  | EU841283 |  |
| *Dasyphyllum argenteum* Kunth |  |  |  |  | AF412855 |
| *Dasyphyllum armatum* (J. Kost.) Cabrera |  |  |  |  | AF412847 |
| *Dasyphyllum brevispinum* Sagást. and M.O. Dillon | EU841076 | EU547644 |  | EU841290 | EU841158 |
| *Dasyphyllum colombianum* (Cuatrec.) Cabrera |  |  |  |  | AF412848 |
| *Dasyphyllum diacanthoides* (Less.) Cabrera |  |  |  |  | EU841156 |
| *Dasyphyllum excelsum* (D. Don) Cabrera |  |  |  |  | EU841155 |
| *Dasyphyllum ferox* (Wedd.) Cabrera | AF412917 |  |  |  | AF412850 |
| *Dasyphyllum inerme* (Rusby) Cabrera |  |  |  |  | AF412843 |
| *Dasyphyllum popayanense* (Hieron.) Cabrera | EU841075 | EU547643 |  | EU841289 | EU841157 |
| *Dasyphyllum vagans* (Gardner) Cabrera |  |  |  |  | AF412858 |
| *Doniophyton anomalum* (D. Don) Kurtz | EU841082 | EU547651 |  | EU841296 | EU841164 |
| *Doniophyton weddelli* Katinas and Stuessy | EU841081 | EU547650 |  | EU841294 | EU841163 |
| *Duseniella patagonica* (O. Hoffm.) K. Schum. |  |  | JF920295 | JF920290 | EF530227 |
| *Fulcaldea laurifolia* Poir. | EU841083 |  |  | EU841298 | EU841165 |
| *Fulcaldea stuessyi* Roque and V.A. Funk |  |  | JF920294 | JF920289 | KF989504 |
| *Huarpea andina* Cabrera | EU841084 | EU547653 |  | EU841299 | EU841167 |
| *Schlechtendalia luzulaefolia* Less. |  |  |  |  | EU841166 |
|  |  |  |  |  |  |
| *Calycera crassifolia* Hicken | EU841096 | EU547628 |  | EU841314 | EU841176 |
| *Mutisia decurrens* Cav. | EU841087 | EU547657 |  | EU841304 | EU841169 |
